# The Effects of the Endocannabinoids Anandamide and 2-Arachidonoylglycerol on Human Osteoblast Proliferation and Differentiation

**DOI:** 10.1371/journal.pone.0136546

**Published:** 2015-09-28

**Authors:** Marie Smith, Richard Wilson, Sally O’Brien, Cristina Tufarelli, Susan I. Anderson, Saoirse Elizabeth O’Sullivan

**Affiliations:** Division of Medical Sciences & Graduate Entry Medicine, School of Medicine, Royal Derby Hospital, University of Nottingham, Derby DE22 3DT, United Kingdom; University of Alabama at Birmingham, UNITED STATES

## Abstract

The endocannabinoid system is expressed in bone, although its role in the regulation of bone growth is controversial. Many studies have examined the effect of endocannabinoids directly on osteoclast function, but few have examined their role in human osteoblast function, which was the aim of the present study. Human osteoblasts were treated from seeding with increasing concentrations of anandamide or 2-arachidonoylglycerol for between 1 and 21 days. Cell proliferation (DNA content) and differentiation (alkaline phosphatase (ALP), collagen and osteocalcin secretion and calcium deposition) were measured. Anandamide and 2-arachidonoylglycerol significantly decreased osteoblast proliferation after 4 days, associated with a concentration-dependent increase in ALP. Inhibition of endocannabinoid degradation enzymes to increase endocannabinoid tone resulted in similar increases in ALP production. 2-arachidonoylglycerol also decreased osteocalcin secretion. After prolonged (21 day) treatment with 2-arachidonoylglycerol, there was a decrease in collagen content, but no change in calcium deposition. Anandamide did not affect collagen or osteocalcin, but reduced calcium deposition. Anandamide increased levels of phosphorylated CREB, ERK 1/2 and JNK, while 2-arachidonoylglycerol increased phosphorylated CREB and Akt. RT-PCR demonstrated the expression of CB_2_ and TRPV1, but not CB_1_ in HOBs. Anandamide-induced changes in HOB differentiation were CB_1_ and CB_2_-independent and partially reduced by TRPV1 antagonism, and reduced by inhibition of ERK 1/2 and JNK. Our results have demonstrated a clear involvement of anandamide and 2-arachidonoylglycerol in modulating the activity of human osteoblasts, with anandamide increasing early cell differentiation and 2-AG increasing early, but decreasing late osteoblast-specific markers of differentiation.

## Introduction

Bone is a dynamic tissue, constantly being remodelled to adapt to changes in mechanical stresses and repair fractures. This occurs via the action of osteoclasts and osteoblasts which resorb and replace bone respectively. Osteoblasts also have a role in affecting the degree of bone resorption. They express receptor activator of nuclear factor kappa-B ligand (RANKL) which stimulates osteoclastogenesis, but also express osteoprotegrin, a soluble ‘decoy’ receptor, which binds to the ligand and therefore inhibits osteoclast activation [1]. Osteoblasts are a key cell type in maintaining bone health and factors affecting their proliferation and differentiation could give new insights into bone disease.

The endocannabinoid system consists of endogenous cannabinoid ligands (endocannabinoids), the receptors at which they act, and the enzymes involved in their synthesis and degradation [2]. The two first identified and most studied endocannabinoids, N-arachidonoylethanolamine (anandamide) and 2-arachidonoylglycerol (2-AG), are associated with wide ranging physiological processes including appetite stimulation, pain modulation and energy expenditure [3,4]. Cannabinoid receptor 1 (CB_1_) and 2 (CB_2_) are the characterised cannabinoid receptors, and anandamide and 2-AG bind to these in the nanomolar range. 2-AG is suggested to be a full agonist, whereas anandamide is considered a partial agonist [2]. Other receptors which endocannabinoids are known to act at include G-protein coupled receptors (GPR55, GPR119), transient receptor potential vanilloids (TRPV1, TRPV4), peroxisome proliferator-activated receptors (PPARα, PPARγ), and various ion channels [5].

Several research groups have shown that the endocannabinoid system is expressed in bone. CB_1_, CB_2_ and TRPV1 have been identified on human osteoclasts [6] and GPR55 is expressed on both human osteoblasts and osteoclasts [7]. Mouse osteoblasts and osteoclasts express CB_1_ [8,9], CB_2_ [8,10], GPR55 [7] and TRPV1 [11]. Studies into the innervation of bone in mice have shown CB_1_ [12] and TRPV1 [13] expression on sympathetic nerve fibres. Both anandamide and 2-AG are detectable in human osteoclasts and osteoblast-like cells *in vitro* [6,14]. The enzymes diacylglycerol lipases alpha and beta [15] and NAPE-phospholipase D [6], which are required for 2-AG and anandamide synthesis respectively, are also expressed in osteoblasts and osteoclasts. Similarly, the degradation enzymes, fatty acid amide hydrolase (FAAH) and monoacylglycerol lipase (MAGL) have been found in human osteoclasts [6,16] and murine osteoblasts [17].

Studies investigating the potential role of the endocannabinoid system in bone have revealed conflicting results. CB_2_ deficient mice display a normal phenotype initially but develop an increased, age-related, gender independent bone loss [18]. CB_1_ deficient mice have been reported to show both high [8] and low bone mass [13]. These discrepancies are suggested to be due to variations in the mouse models used [13,18]. Synthetic cannabinoid antagonists decrease murine osteoblast [11] and osteoclast function *in vitro* [8,10,11,19] and reduce bone loss associated with an ovariectomy in mice *in vivo* [8,10,11,19,20], implying a role for endogenous agonists of these receptor in stimulating bone growth. However, there have been few studies on the direct effects of the endocannabinoids themselves. *In vitro* 2-AG treatment of rat bone marrow stromal cell (BMCs) increases alkaline phosphatase (ALP) and collagen, markers of osteoblast differentiation [21]. In contrast, Tam *et al*. [15] found 2-AG to decrease ALP production and cell number of a murine osteoblast cell line (MC3T3-E1). Idris *et al*. [8] showed anandamide treatment stimulated osteoclast formation in murine osteoclast cultures, although another study showed that anandamide and 2-AG reduce osteoclast formation through inhibition of differentiation [14]. To date, no studies have examined the effects of endocannabinoids on human osteoblast (HOB) growth. Therefore, the aim of the present study was to investigate the direct effects of the endocannabinoids anandamide and 2-AG on the proliferation and differentiation of HOBs *in vitro*.

## Methods

### Human osteoblast culture

Human osteoblasts (HOBs) were originally isolated from human femoral head trabecular bone and were characterised previously at equivalent passages and time-periods [22–25]. Cells were seeded in 24 well plates at 8 x 10^4^ cells/well. They were fed every 2–3 days with Dulbecco’s Modified Eagle’s Medium supplemented with 10% FBS, 1% penicillin and streptomycin, 15μg.ml^-1^ ascorbic acid (aids collagen matrix formation [26]), 10nM dexamethasone (helps to maintain osteoblast phenotype and enhance differentiation [27,28]), 10mM sodium β-glycerophosphate (source of inorganic phosphate for matrix mineralisation [29]) and 200nM L-glutamine (all Sigma, UK).

### Endocannabinoid treatment protocol

From the time of seeding, cells were treated with medium supplemented with 1nM, 10nM, 100nM, 1μM, 10μM anandamide (Tocris, UK) or 2-arachidonoylglycerol (Ascent, UK) or their vehicle, ethanol (0.1%). Cells were treated with the endocannabinoids for 24 hours, 4 days (to capture the proliferative phase of HOB growth and the early markers of differentiation) or 21 days (for later markers of differentiation such as matrix elaboration and calcification). After the treatment protocols, the media was removed and stored at -80°C, and the cells were lysed by 3 cycles of freeze-thawing in 1ml distilled water and stored at -80°C for later analysis.

To increase local levels of endocannabinoid, HOBs were treated for 24 hours with endocannabinoid degradation enzyme inhibitors, or vehicle (0.1% ethanol and 0.1% DMSO). Media was supplemented with 1μM URB597 (Sigma, UK), a fatty acid amide hydrolase inhibitor, an enzyme that degrades anandamide, or 1μM JZL184 hydrate (Sigma, UK), a monoacylglycerol lipase inhibitor, an enzyme that degrades 2-AG.

### Proliferation and differentiation analysis

DNA content was measured by incubating cell lysate samples for 5–10 seconds with 1% bisBenzimide H (Hoechst 33258) dye (Sigma, UK). Samples were measured in duplicate and the fluorescence was read at excitation 355nm and emission 460nm using a Fluoroskan Ascent reader (Thermo Scientific, USA) and interpolated from a standard curve of calf thymus DNA (Sigma, UK).

Alkaline phosphatase (ALP) activity was measured using p-Nitrophenyl phosphate (Sigma, UK) which forms a complex with ALP present in the cell lysate. The samples were incubated for 10 minutes and the resulting substrate had an absorbance which was read, in duplicate, at 405nm.

Intact and N-terminal—mid fragment osteocalcin levels were measured in the media collected at 4 days using an N-MID Osteocalcin ELISA (Immuno Diagnostic Systems, UK). The resulting chromogenic substrate was measured at 450nm with 650nm as a reference and the measurements were interpolated based on a standard curve of synthetic human osteocalcin.

Calcium production was measured at the end of the 21 day culture period. 1% (*w/v*) alizarin red S (Sigma, UK) was added to the wells and left to form a chelate complex with calcium for 10 minutes. The dye was dissociated with 4% (*w/v*) cetylpyridinium chloride monohydrate (Sigma, UK) and the resulting solution absorbance was measured, in duplicate, at 562nm [30].

Collagen was measured in cell lysates using the Sircol collagen assay kit (Biocolor, UK). Samples were measured in duplicate at 540nm and interpolated from a standard curve of rat collagen (Biocolor, UK). Absorbances for all assays were measured using a multiskan spectrum spectrophotometer (Thermo Scientific, USA).

### Cell signalling analysis

HOBs were cultured for 24 hours and then treated with vehicle (0.1% ethanol), 10μM anandamide or 10μM 2-AG for 20 minutes. The cells were lysed in RIPA buffer (Roche Applied Science, UK), and the supernatant was used for signalling protein analysis. Levels of phosphorylated signalling proteins (ERK/MAP kinase 1/2, Akt, STAT3, JNK, p70 S6 kinase, NFκB, STAT5 A/B, CREB and p38) were measured using a Milliplex map multi-pathway magnetic bead 9-plex signalling multiplex assay kit (48-680MAG, Millipore, USA). The plate was read using a MAGPIX analyser (Luminex, USA) and sample values were interpolated from known standards using the built-in xPONENT software. All results were normalised to the protein content from the corresponding sample as measured using a bicinchoninic acid protein assay. A 1:50 ratio of copper (II) sulphate pentahydrate 4% solution: bicinchoninic acid (Sigma, UK) was added to each sample and incubated at 37°C on a shaker. Samples were measured in duplicate at 562nm and interpolated from a standard curve of bovine serum albumin (Sigma, UK).

### RT-PCR

The presence of predicted sites of action was investigated at the mRNA level using reverse transcription followed by polymerase chain reaction (RT-PCR). Hypoxanthine-guanine PhosphoRibosylTransferase was used as the control house-keeping gene, and human astrocytes (HAs) were used as a positive control known to express the target sites of action of interest; CB_1_R, CB_2_R and TRPV1 [31]. RT-PCRs for the various target genes analysed were performed as previously described by Hind *et al*. [31].

### Effect of cannabinoid receptor antagonists and intracellular signalling inhibitors

Based on previous results, HOBs were treated with specific signalling inhibitors and receptor antagonists to identify any changes in the cell response to anandamide. HOBs were treated with media supplemented with 100nM AM251 (CB_1_R antagonist), 100nM AM630 (CB_2_R antagonist), 1μM capsazepine (TRPV1 antagonist), 500nM KT5720 (PKA inhibitor, PKA is upstream of CREB), 1μM PD98059 (MEK inhibitor) or 10μm SP600125 (JNK inhibitor) for 30 minutes before the addition of vehicle (0.1% ethanol) or 10μM anandamide (all Tocris, UK). HOBs were also treated with media supplemented with 10μM anandamide only and vehicle only (0.1% ethanol and 0.1% DMSO).

### Statistical analysis

Data was calculated as a percentage change compared to the average vehicle measurements in a given experiment. Data presented as mean ± SEM with sample groups of *n* = 6–16 from 2–4 separate experiments. Group comparisons were analysed by one-way ANOVA with Dunnett’s *post-hoc* test compared to the vehicle using Prism (Graphpad, USA). Comparison of drug treatments was analysed by two-way ANOVA using Prism.

## Results

### Temporal changes in HOB proliferation and differentiation *in vitro*

**Fig 1:**
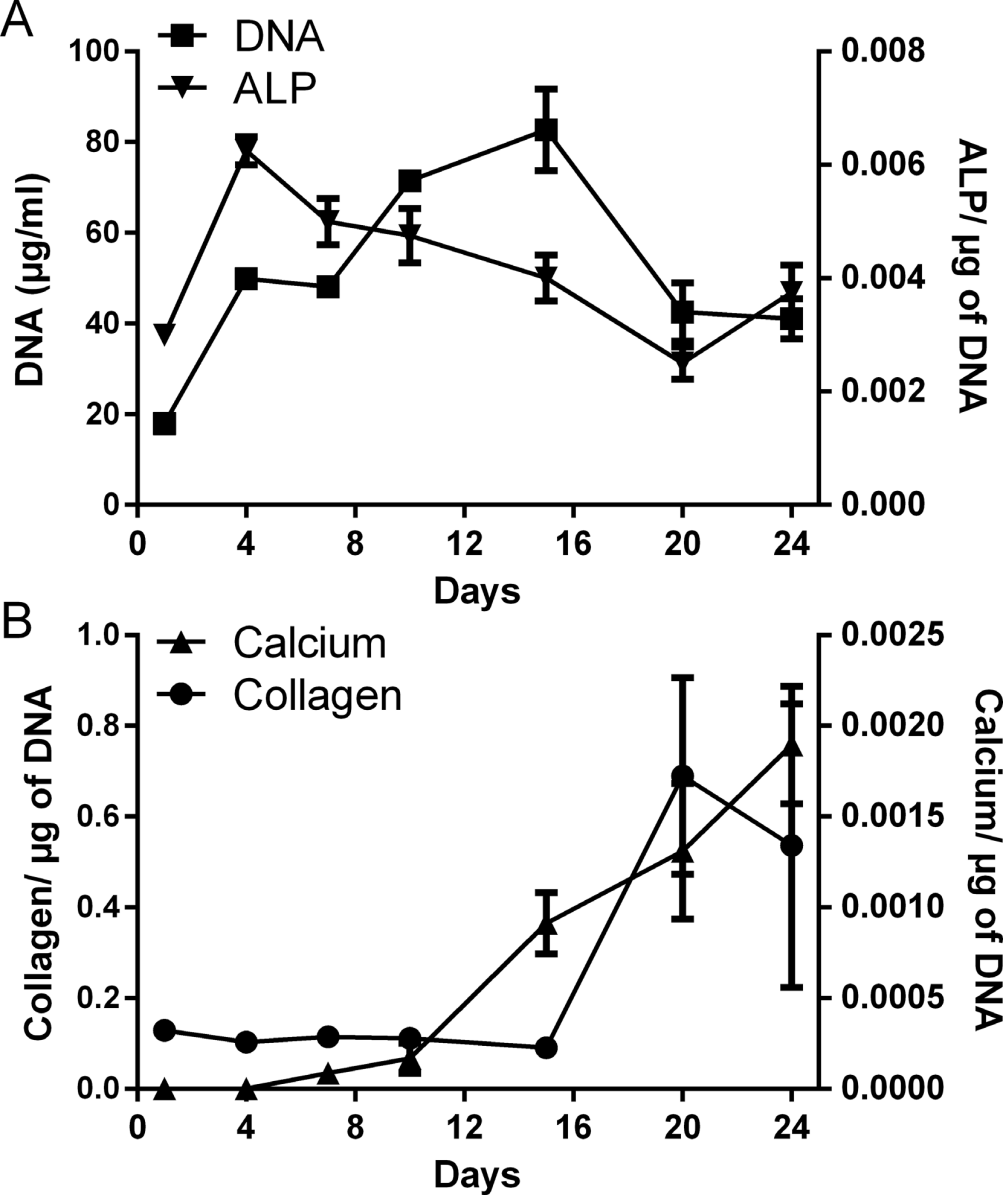
Temporal changes in HOB proliferation and differentiation *in vitro*. (A) DNA content and ALP production, and (B) collagen and calcium production. Values normalised to DNA content. Data given as mean ± S.E.M., n = 4, from 1 experiment.

HOBs were cultured for up to 24 days to establish the normal temporal changes in osteoblast proliferation and differentiation. HOB cultures were found to mature over a 3–4 week period, producing a range of markers consistent with the phases of proliferation, organic matrix production (osteoid), and matrix maturation (calcification) [32]. DNA content increased until day 15, after which, it decreased and plateaued between days 20 and 25 (Fig 1A). ALP activity peaked at day 4, and then showed a gradual decrease until day 15 (Fig 1A). Calcium production was apparent from day 15 and increased continually until day 25 (Fig 1B). Collagen production was present at a low level from day 1 but increased from day 20 (Fig 1B).

### Effect of anandamide and 2-AG treatment on HOB proliferation and markers of early differentiation

**Fig 2:**
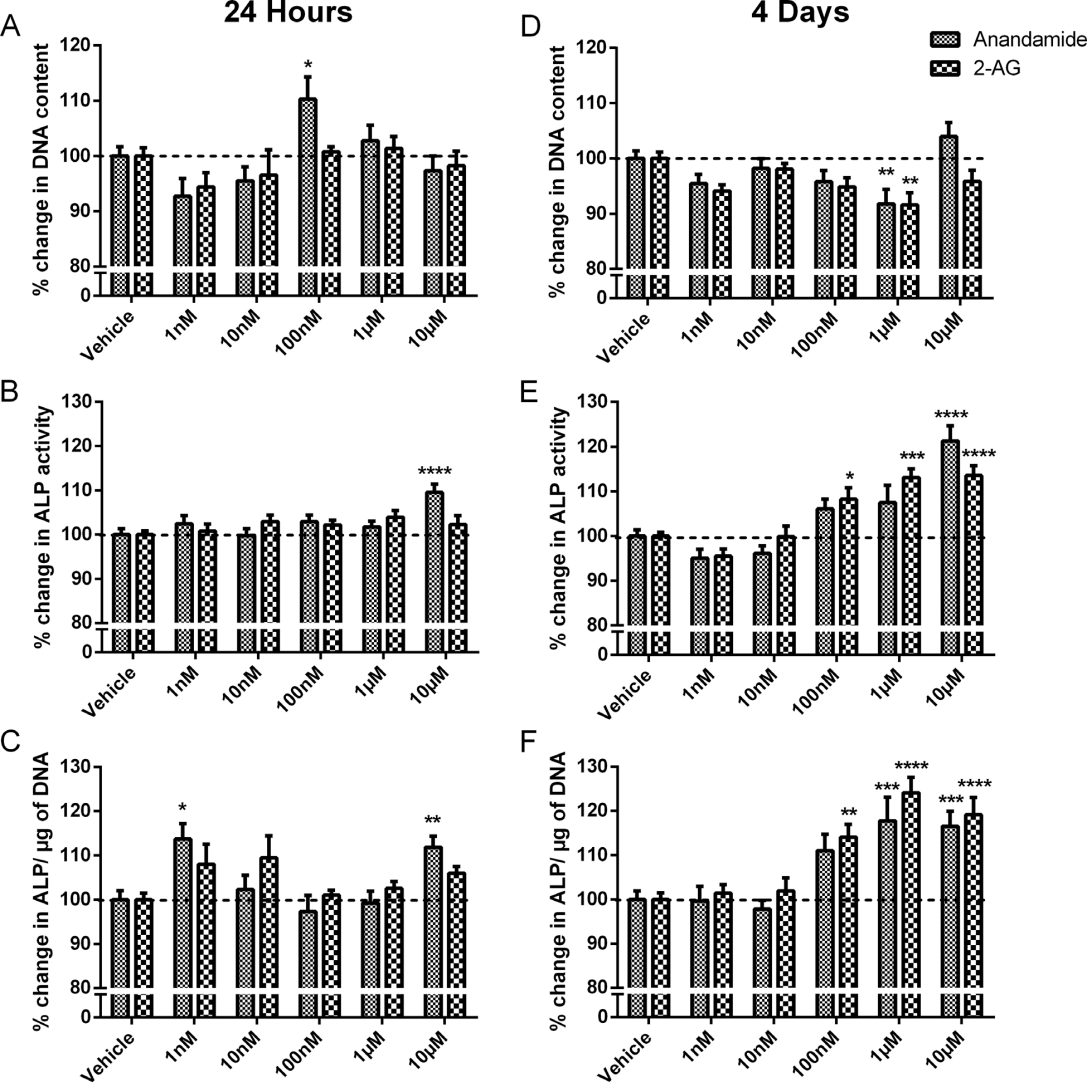
Effect of anandamide and 2-AG treatment on HOB proliferation and markers of early differentiation. DNA content of cells treated with increasing concentrations of anandamide and 2-AG after (A) 24 hours and (D) 4 days. ALP production of treated cells after (B) 24 hours and (E) 4 days, and ALP production normalised to DNA content after (C) 24 hours and (F) 4 days. Values are expressed as a percentage of the vehicle, dotted line at 100%. Data given as mean ± S.E.M., *n* = 15–16, from 4 experiments. * *P*<0.05, ** *P*<0.01, *** *P*<0.001, **** *P*<0.0001; one—way ANOVA with Sidak’s multiple comparisons test.

Treatment with anandamide at 100nM, but not other concentrations, for 24 hours significantly increased DNA content (*P*<0.05, Fig 2A), and 1μM anandamide treatment for 4 days, significantly decreased DNA content (*P*<0.05, Fig 2D). After 24 hours or 4 days treatment with 10 μM anandamide, HOBs had significantly increased ALP activity compared to vehicle (*P*<0.0001, Fig 2B and 2E). When normalised to DNA content, ALP production was significantly increased in cells treated for 24 h (at 1 nM and 10 μM) or 4 days (at 1 and 10 μM) with anandamide (Fig 2C and 2F).

24 hour treatment with 2-AG had no significant effect on DNA or ALP. 4 days treatment with 1μM 2-AG significantly decreased DNA content (P<0.01, Fig 2D) and resulted in a concentration-dependant increase in ALP activity (from 100nM to 10μM, *P*<0.05-*P*<0.0001, Fig 2E). Normalising ALP production to DNA content maintained this effect (*P*<0.01-*P*<0.0001, Fig 2F).

There was no statistical difference when comparing the effects of anandamide and 2-AG on changes in DNA content or ALP production in HOBs, as measured by two-way ANOVA.

### Effect of anandamide and 2-AG treatment on late differentiation markers of HOBs

**Fig 3:**
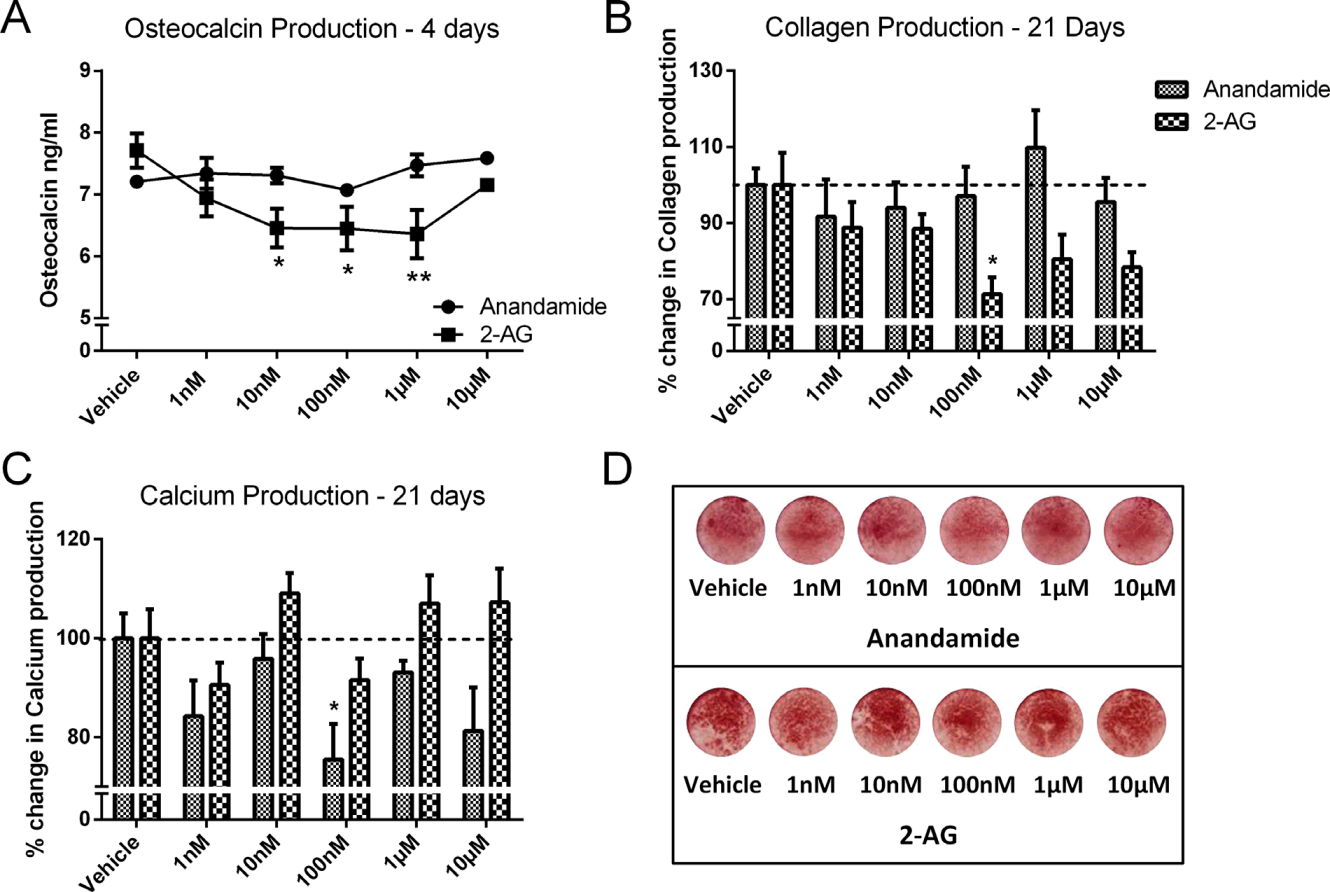
Effect of anandamide and 2-AG treatment on late differentiation markers of HOBs. (A) Osteocalcin production in cells treated with increasing concentrations of anandamide or 2-AG for 4 days, and (B) collagen production of treated cells after 21 days. (C) Mean quantative analysis of calcium production of cells after 21 days treatment, as measured by alizarin red S staining, and (D) representative images of alizarin red S stained cells, showing calcium deposits. Values are expressed as a percentage of the vehicle, dotted line at 100%. Data given as mean ± S.E.M., *n* = 4–10, from 1–3 experiments. * *P*<0.05, ** *P*<0.01; one—way ANOVA with Sidak’s multiple comparisons test.

Anandamide treatment had no effect on osteocalcin secretion from HOBs. By contrast, HOBs treated with 10 nM-1 μM 2-AG for 4 days, had significantly decreased osteocalcin production (*P*<0.05-*P*<0.01, Fig 3A). Collagen and calcium production were minimal before 15–20 days in the preliminary experiment (see Fig 1B). Therefore, we examined the effects of anandamide and 2-AG treatment on these measures after 21 days. Treatment of HOBs with 100nM 2-AG for 21 days resulted in a significant decrease in collagen production (*P*<0.05, Fig 3B), however, 2-AG treatment had no effect on calcium production at the same time-point. After 21 days, cells treated with 100nM anandamide had significantly reduced calcium production (*P*<0.05, Fig 3C), but anandamide treatment had no effect on the collagen production of the cells. Representative images of the cells treated with both anandamide and 2-AG showed clear alizarin red S labelling of calcium deposits after 21 days (Fig 3D).

### Effect of FAAH and MAGL inhibitors on HOB proliferation and differentiation

**Fig 4:**
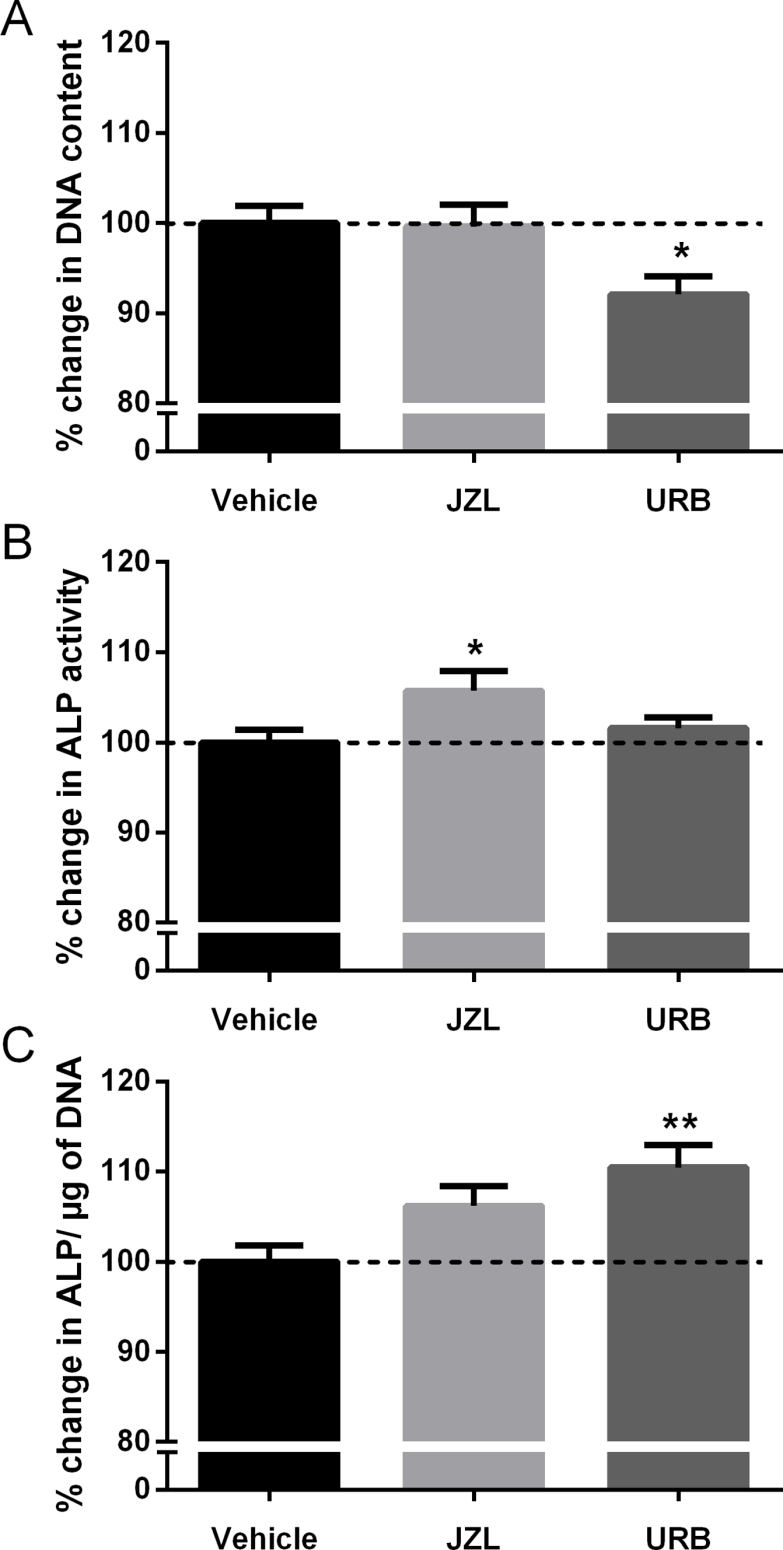
Effect of FAAH and MAGL inhibitors on HOB proliferation and differentiation. (A) DNA content of cells treated with 1μM JZL184 hydrate (JZL, a MAGL inhibitor) and 1μM URB597 (URB, a FAAH inhibitor) for 24 hours. (B) ALP production of treated cells, and (C) ALP production normalised to DNA content. Values are expressed as a percentage of the vehicle, dotted line at 100%. Data given as mean ± S.E.M., *n* = 8, from 2 experiments. * *P*<0.05, ** *P*<0.01; one—way ANOVA with Dunnett’s multiple comparisons test.

HOBs were treated with endocannabinoid degradation enzyme inhibitors to assess whether similar changes in HOB function could be observed by increasing the endogenous concentrations of anandamide and 2-AG. URB597, a FAAH inhibitor which would increase endogenous levels of anandamide, significantly decreased DNA content (*P*<0.05, Fig 4A), and increased ALP production (*P*<0.01, Fig 4C), when normalised to DNA content. MAGL is involved in the degradation of 2-AG, and its inhibitor, JZL184 hydrate, significantly increased the ALP production of HOBs (*P*<0.05, Fig 4B), but had no effect on DNA content.

### Effect of anandamide or 2-AG treatment on intracellular signalling in HOBs

**Fig 5:**
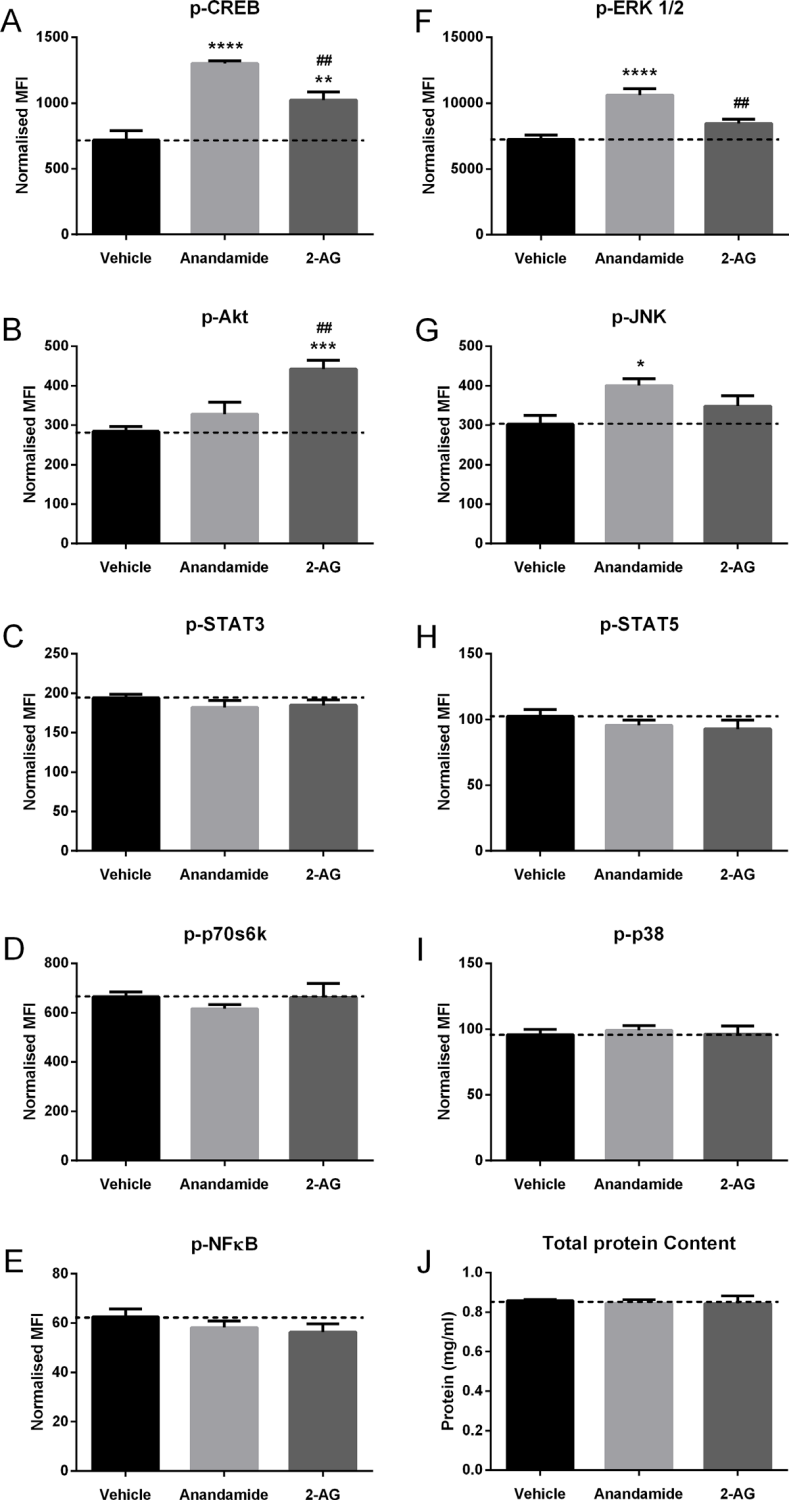
Effect of anandamide or 2-AG treatment on intracellular signalling in HOBs. Cells were grown for 24 hours *in vitro*, then treated with vehicle, 10μM anandamide or 10μM 2-AG for 20 minutes and multiplex analysis used to measure the level of phosphorylated (A) CREB, (B) Akt, (C) STAT3, (D) p70s6k, (E) NFκB, (F) ERK 1/2, (G) JNK, (H) STAT5 and (I) p38 expressed as mean fluorescence intensity (MFI) normalised to the (J) protein content of each sample. Data given as mean ± S.E.M., *n* = 6, from 1 experiment. * *P*<0.05, ** *P*<0.01, *** *P*<0.001, **** *P*<0.0001, compared to vehicle; # # *P*<0.01, compared to anandamide; one—way ANOVA with Tukey’s multiple comparisons test.

To establish the intracellular signalling pathways that might be activated by endocannabinoids in HOBs, confluent cells were treated with 10μM anandamide or 10μM 2-AG for 20 minutes, and the cells were analysed for changes in levels of phosphorylated signalling molecules. The signalling proteins was normalised to total protein content for each sample, which was consistent for each treatment group (Fig 5J). Anandamide significantly increased the levels of phosphorylated (p)-CREB (*P*<0.0001, Fig 5A), p-ERK 1/2 (*P*<0.0001, Fig 5F) and p-JNK (*P*<0.05, Fig 5G). 2-AG increased the levels of p-Akt (*P*<0.001, Fig 5B) and p-CREB (*P*<0.01, Fig 5A) compared to the cultures treated with vehicle. Although both endocannabinoids significantly increased the levels of p-CREB, osteoblasts treated with anandamide had significantly higher levels than those treated with 2-AG (*P*<0.01, Fig 5A). Neither endocannabinoid had an effect on the levels of p-NFκB, p-p38, p-p70s6k, p-STAT3 or p-STAT5 (Fig 5).

### Effect of anandamide on HOB proliferation and differentiation in the presence of cannabinoid receptor antagonists and intracellular signalling inhibitors

**Fig 6:**
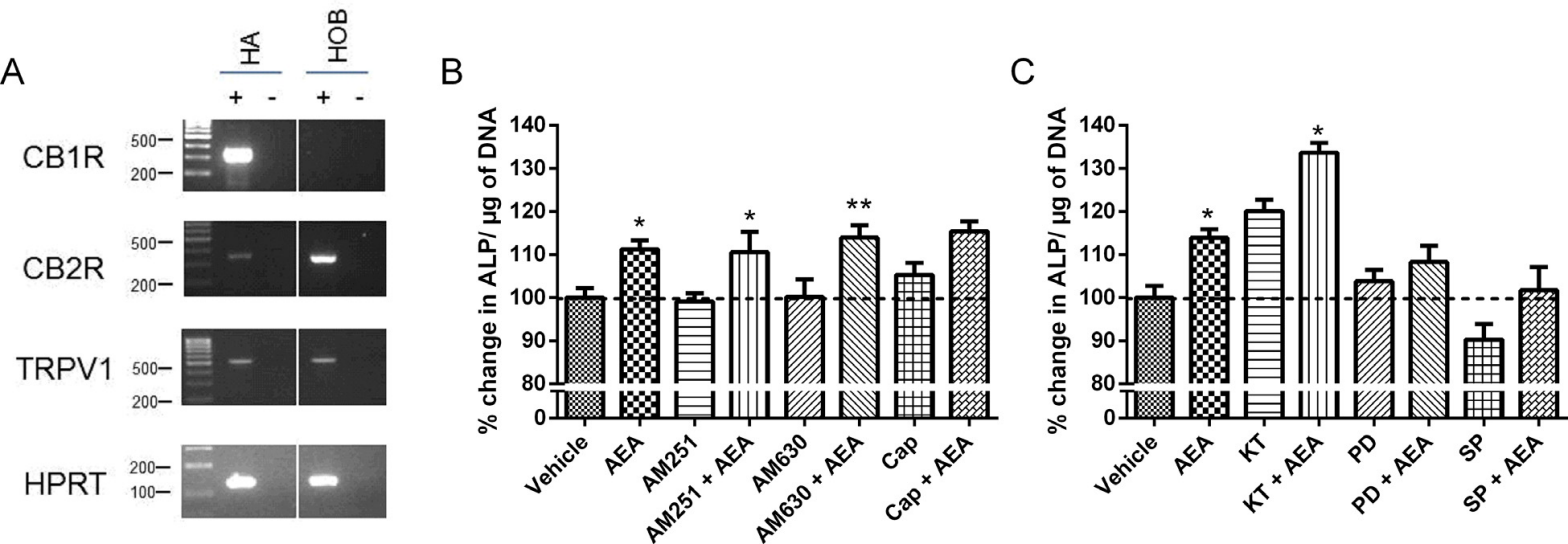
Effect of anandamide on HOB proliferation and differentiation in the presence of cannabinoid receptor antagonists and intracellular signalling inhibitors. (A) RT-PCR analysis of cannabinoid receptor mRNA expression in HOBs. Human astrocytes (HA) were used as a positive control for CB1R, CB2R and TRPV1 expression. Hypoxanthine-guanine PhosphoRibosylTransferase (HPRT) was used as the control house-keeping gene. (B) ALP production, normalised to DNA content, was measured in HOBs grown for 4 days and treated with 10μM anandamide (AEA) only, or 10μM anandamide and the following antagonists and inhibitors. Cells were treated with AM251 (100nM, CB_1_R antagonist), AM630 (100nM, CB_2_R antagonist) or Capsazepine (1μM, Cap, TRPV1 antagonist) for 30 minutes before the addition of vehicle or anandamide. (C) Cells were treated with KT5720 (500nM, PKA inhibitor), PD98059 (1μM, MEK inhibitor) or SP600125 (10μm, JNK inhibitor) for 30 minutes before the addition of vehicle or anandamide. Data given as mean ± S.E.M., *n* = 9, from 3 experiments. * *P*<0.05, ** *P*<0.01, compared to antagonist/inhibitor only; one-way ANOVA with Sidak’s multiple comparisons test.

To gain insights into the molecular basis for the observed pharmacological findings, RT-PCR was used to investigate the mRNA expression of CB_1_R, CB_2_R and TRPV1 in HOBs. Human astrocytes were previously shown to express all of the targets [33] and so used as a positive control. HOBs were found to express CB_2_R and TRPV1 but not CB_1_R (Fig 6A).

Anandamide treatment increased normalised ALP production (*P*<0.05, Fig 6B and 6C) compared to vehicle. Anandamide also increased normalised ALP production in the presence of AM251 (CB_1_R antagonist, *P*<0.05) and AM630 (CB_2_R antagonist, *P*<0.01), but not in the presence of capsazepine (TRPV1 antagonist, Fig 6B). Anandamide treatment increased normalised ALP production in the presence of KT5720 (PKA inhibitor, *P*<0.05), but not the presence of PD98059 (MEK inhibitor) or SP600125 (JNK inhibitor, Fig 6C).

## Discussion

The endocannabinoid system appears to play a role in the modulation of bone cells, but there is conflicting evidence in the literature as to whether this is pro-osteogenic or anti-osteogenic. There is also limited research into the effects of endocannabinoids on human osteoblasts, which was the aim of the present study. Our results have demonstrated a clear involvement of two endocannabinoids in modulating the activity of human osteoblasts, with anandamide increasing early cell differentiation and 2-AG increasing early, but decreasing late osteoblast-specific markers of differentiation.

### Effects of anandamide on osteoblast function

Anandamide decreased the proliferation of HOBs at early time-points. Despite this, the highest concentrations of anandamide increased osteoblast differentiation, as measured by alkaline phosphatase (ALP) production. The validity of these results was confirmed when similar results were obtained using URB597, a FAAH inhibitor, which would act to increase endogenous levels of anandamide. ALP is essential for bone mineralisation as it increases the local concentration of phosphate ions and aids in the construction of hydroxyapatite crystals and the mineralised matrix [22]. Together our data suggest that anandamide decreases proliferation but increases differentiation, which might lead to increased osteoblast mineralisation. However, treatment with anandamide for longer time periods (21 days) had a negative effect on calcium production, a late marker of differentiation. Only one study to date has looked at the direct effects of anandamide on osteoblasts and found that treatment of primary mouse osteoblasts with up to 20μM anandamide resulted in no change in cell growth [8]. This could be due to differences in the cell origin species, or in and treatment methodology. In the present study cells were treated with anandamide from the time of seeding, while Idris et al. treated confluent cells. It is likely that the expression of the endocannabinoid system and target sites of action changes during cell maturation [17,34].

PCR demonstrated the expression of CB_2_ and TRPV1 but not CB_1_ in our HOB cells, which fits with the current literature [10,11,18]. We found that the effects of anandamide on ALP were unaffected by CB_1_ or CB_2_ antagonism, but were no longer significant in the presence of a TRPV1 antagonist, suggesting a partial role for this receptor in osteoblast activity, as previously demonstrated [11]. The endocannabinoid system has been linked with common bone disorders including osteoporosis and osteoarthritis. In a clinical study, polymorphisms in the CB_2_R gene in humans were linked with the onset of osteoporosis [35]. A similar effect was observed in CB_2_R KO mice, which display a normal phenotype initially, but start to display increased age related, gender independent, bone loss [18]. CB_2_ has been proposed as a target in osteoporosis [36] and rheumatoid arthritis [37]

Further investigation into potential pathways of action showed that anandamide increased phosphorylated CREB, ERK 1/2 and JNK in HOBs. Jaiswal *et al*. [38] showed ERK 1/2 to be associated with early bone differentiation including ALP production, and an increase in ALP production was found in the osteoblasts of ERK 1/2 over-expressing mice [39], suggesting activation of ERK is a possible mechanism by which anandamide increases early differentiation. As confirmation of this, we found that anandamide was no longer able to increase ALP in the presence of an ERK inhibitor. The CREB pathway has been linked to differentiation, with an increase in ALP activity being observed in murine osteoblasts over-expressing CREB [40], however we did not observe an effect of inhibiting PKA upstream of CREB (inhibitor of CREB are not commercially available). JNK signalling has been shown to have a negative effect on ALP activity and calcium production in a murine osteoblastic cell line [41], and mouse bone marrow stromal cells [42] although promotes osteogenic differentiation in human mesenchymal cells [43,44]. We found that inhibiting JNK decreased differentiation in HOBS, and although there was still a stimulatory effect of anandamide in the presence of a JNK inhibitor, it was no longer significant, suggesting this pathway is also involved in anandamide’s effect on HOBs.

### Effects of 2-AG on osteoblast function

The effects of 2-AG treatment were all observed at the 4 day time-point. As seen with anandamide treatment, DNA content was decreased and ALP production was increased. Unlike anandamide, this was observed in both the high nanomolar and micromolar range. Similar increases in ALP activity was seen after treatment with JZL184 hydrate, an inhibitor of the 2-AG degrading enzyme MAGL, which would increase endogenous 2-AG levels. The decrease in DNA content observed is consistent with findings by Tam *et al*. [15] who found no effect on cell number of MC3T3-E1 cells treated with similar concentrations of 2-AG. However, they found no change in ALP activity [15]. However, in agreement with our results, another study using bone marrow stromal cells found treatment with similar concentrations of 2-AG resulted in a significant increase in ALP producing cells [21].

Osteocalcin is secreted by osteoblasts and activated during bone remodelling, with the active form having a role in the production and release of insulin via activation of pancreatic cells (see [45] for a review). There is also evidence for osteocalcin having a role in the negative regulation of bone formation. Ducy *et al*. [46] found osteocalcin-deficient mice had an increased amount of mineralised bone matrix, and the formation rates of their cancellous and cortical bone were increased. Therefore, a decrease in the production of osteocalcin by osteoblasts, as observed in cells treated with 2-AG, may have a positive effect on bone mineralisation and growth *in vivo*.

The present study also demonstrated that 2-AG had a negative effect on collagen production, a marker of late HOB differentiation. Collagen is the main component of the extracellular matrix. It is assembled into fibrils outside the cell, after release of procollagen from osteoblasts, and hydroxyapatite crystals bind to these organised fibrils during mineralisation. We have therefore demonstrated that 2-AG reduces the production of the scaffold upon which new bone is laid implying that *in vivo*, this would equate to reduced production or weakening of bone matrix. In contrast to our findings, Scutt and Williamson [21] found an increase in collagen production of rat primary bone marrow cells using the same 2-AG concentration range. However, in addition to the difference in cell type, the cells were collected after 15 days, with the drug treatment occurring for only the first 5 days. In comparison, the present study investigated cells which were cultured up to 21 days and were treated with 2-AG from the time of seeding, and throughout the experiment.

2-AG increased phosphorylated CREB and Akt in treated HOBs. Increased CREB signalling has been associated with bone metabolism [40] and osteoblast-specific CREB knock-out mice have been shown to have decreased bone volume compared to wild-type animals [47]. There is also a link between Akt signalling and bone. Peng *et al*. [48] observed that Akt1 and Akt2 double KO mice have delayed ossification, due to a decrease in osteoblast activity. In addition, Mukherjee and Rotwein [49] demonstrated the importance of Akt in positively regulating osteoblast differentiation, and specifically ALP and calcium production, and expression of osteocalcin. This suggests that Akt is a potential mechanism for the increased ALP production observed in the 2-AG treated HOBs in the present study, but is not consistent with the decrease in osteocalcin observed.

### Comparison of the effect of anandamide versus 2-AG

The present study has shown anandamide and 2-AG to have similar effects on the early phases of the maturation of HOBs, with both increasing ALP as an early marker. However, they appear to have different roles in the later stages of matrix maturation, with anandamide decreasing calcium and 2-AG reducing collagen and osteocalcin. The signalling protein expression data highlight differences in the pathways each endocannabinoid activates.

In summary, these data provide evidence for a direct peripheral action of 2-AG and anandamide on human osteoblasts. This study shows, for the first time, that these endocannabinoids have a time-dependent effect on the differentiation of human osteoblasts. In an *in vivo* environment; this would translate to increased levels of the endocannabinoids having an initial positive effect on the mineralisation of the bone matrix, however, extended periods of heightened levels could result in a poorly structured and mineralised bone.
